# Molecular Subtyping and Therapeutic Targeting of IFNG‐Driven Immunogenic Cell Death in Lung Adenocarcinoma

**DOI:** 10.1002/cam4.70678

**Published:** 2025-02-13

**Authors:** Lifeng Li, Yaqi Yang, Mengle Peng, Biyue Wang, Lili Zhu, Chengxin Chen, Zhirui Fan, Xiaoran Duan, Ruyue Xue, Xuefeng Lv, Ming Cheng, Jie Zhao

**Affiliations:** ^1^ National Engineering Laboratory for Internet Medical Systems and Applications, the First Affiliated Hospital of Zhengzhou University Zhengzhou University Zhengzhou Henan China; ^2^ Cancer Center, the First Affiliated Hospital of Zhengzhou University Zhengzhou University Zhengzhou Henan China; ^3^ Medical School Huanghe Science and Technology University Zhengzhou Henan China; ^4^ Fuwai Central China Cardiovascular Hospital Internet Medical and System Applications of National Engineering Laboratory Zhengzhou Henan China; ^5^ Department of Clinical Laboratory Henan No. 3 Provincial People's Hospital Zhengzhou Henan China; ^6^ College of Public Health Zhengzhou University Zhengzhou China; ^7^ Department of Nephrology Seventh People's Hospital of Zhengzhou Zhengzhou Henan China; ^8^ Department of Pharmacy, the First Affiliated Hospital of Zhengzhou University Zhengzhou University Zhengzhou Henan China; ^9^ Department of Integrated Traditional and Western Medicine, the First Affiliated Hospital of Zhengzhou University Zhengzhou University Zhengzhou Henan China; ^10^ Department of Clinical Laboratory The Third Affiliated Hospital of Zhengzhou University Zhengzhou Henan China; ^11^ Department of Medical Information, the First Affiliated Hospital of Zhengzhou University Zhengzhou University Zhengzhou Henan China

**Keywords:** IFNG, immunogenic cell death, lung adenocarcinoma, molecular subtypes, tumor microenvironment

## Abstract

**Background:**

Immunogenic cell death (ICD) can be triggered by various therapies to induce anti‐tumor immune responses, significantly enhancing treatment effectiveness, and is widely utilized in tumor immunotherapy.

**Methods:**

LUAD data from The Cancer Genome Atlas (TCGA) and Gene Expression Omnibus (GEO) validated ICD‐related molecular subtypes via consensus clustering. Clinical features, ICD genes, driver genes, mutations, tumor microenvironment, immune checkpoints, and drug sensitivity were compared. RT‐qPCR, Western blot, immunofluorescence, ELISA, flow cytometry, and tube formation assays validated findings.

**Results:**

Differential expression of 33 ICD genes was observed between tumor and normal tissues. These genes were clustered into two groups via consensus clustering and validated with GEO data. Prognostic analysis indicated superior outcomes in cluster 2 across TCGA and GEO cohorts. Significant disparities in clinicopathological characteristics like stage, gender, and age were noted between subtypes. Cluster 2 exhibited heightened expression of ICD‐related genes, driver genes, immune checkpoints, and immune cells. Cluster 2 also showed increased sensitivity to chemotherapy drugs. IFNG overexpression in A549 and H1299 cells induced CRT exposure, HMGB1 release, and ATP secretion, thereby promoting dendritic cell maturation and enhancing CD8+ T cell function. Additionally, IFNG boosted tumor angiogenesis via HMGB1 pathways, which could be mitigated by HMGB1 inhibition.

**Conclusion:**

Identification of novel ICD‐related molecular subtypes holds promise for guiding personalized therapies, assessing prognosis, and predicting immunotherapy efficacy in LUAD. IFNG emerges as a potential prognostic biomarker and therapeutic target, influencing both the tumor microenvironment and angiogenesis. These findings offer new insights into therapeutic strategies targeting IFNG‐mediated pathways in LUAD.

## Graphical abstract

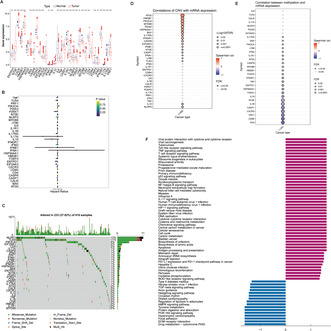

## 1 Introduction

Lung cancer ranks second globally in incidence and is the leading cause of cancer‐related deaths as of 2020 [1]. Lung adenocarcinoma (LUAD), the predominant subtype, exhibits a five‐year survival rate below 20%, despite advances in treatment modalities [2, 3, 4]. Traditional histological classifications face challenges in LUAD management due to its complexity and heterogeneity [5]. Consequently, molecular subtype exploration has gained traction. For instance, Zhang et al. identified three ferroptosis‐related LUAD subtypes based on 14 genes, correlating with immunotherapy efficacy [6]. Similarly, Liu et al. categorized LUAD patients into three pyroptosis‐related subtypes, revealing varied prognostic outcomes [7]. Despite advancements, improving overall survival (OS) in LUAD necessitates identifying new prognostic subtypes for personalized treatments.

Immunogenic cell death (ICD), a regulated process, stimulates adaptive immune responses [8, 9]. ICD is triggered by various stimuli including pathogens, chemotherapy (anthracyclines, oxaliplatin), targeted drugs, radiotherapy, irradiation, and photodynamic therapy [10]. During ICD, molecules known as damage‐associated molecular patterns (DAMPs) are released or exposed from stressed or dying cells [11, 12]. Common DAMPs involved in ICD include ATP, high mobility group protein B1 (HMGB1), calreticulin (CRT), heat shock protein 90, and uric acid, which serve as biomarkers [13]. Through external stimuli, dying or dead cancer cells undergo a transformation from non‐immunogenic to immunogenic states, bolstering anticancer immunity [14].

Several approved drugs induce ICD and enhance anti‐cancer immunity: Belantamab Mafodotin for multiple myeloma, Lurbinectedin for small cell lung cancer [15]. Additionally, certain cytotoxic chemotherapeutic agents have been identified as ICD inducers [16]. For example, oxaliplatin can induce ICD effectively in colorectal cancer [17], whereas cisplatin lacks CRT exposure induction but enhanced ER stress can restore it [18, 19]. Immunotherapy advancements prompt interest in enhancing efficacy via ICD induction. Current research predominantly explores the impact of nanotechnology [20], ultrasound [21], and immune checkpoint blockade [22] on ICD, yet studies focusing on ICD‐related gene regulation remain limited. Optimizing outcomes, especially in LUAD patients, necessitates novel investigative strategies.

In this study, utilizing 33 identified ICD‐related genes, we stratified patients into two subtypes and validated our findings, revealing significant differences in clinical characteristics, prognosis, gene mutations, tumor microenvironment (TME), immune checkpoint expression, and drug sensitivity between the subtypes. Investigation into the ICD‐related gene interferon gamma (IFNG) showed that its overexpression in LUAD cells enhances CRT exposure, promotes HMGB1 and ATP release, strengthens ICD, facilitates dendritic cell (DCs) maturation, enhances CD8^+^ T cell function, and promotes tumor angiogenesis dependent on HMGB1.

## 2 Materials and Methods

### 2.1 Data Collection and Analysis

We retrieved transcriptomic and clinical data concerning LUAD from The Cancer Genome Atlas (TCGA), and 33 ICD‐related genes were identified. Validation was performed using the Gene Expression Omnibus (GEO) datasets (GSE31210, GSE50081, GSE37745, GSE68465), which underwent batch correction using the SVA package.

### 2.2 Molecular Mechanism and Pathway Analysis of ICD‐Related Genes

The 33 genes associated with ICD underwent analysis for mutations, copy number variations (CNV), and methylation. We utilized single‐sample gene set enrichment analysis (ssGSEA) to assign scores to each sample according to these genes, while also examining their correlations with the entire gene set. Pathway analysis focused on genes showing significant positive and negative correlations.

### 2.3 Construction and Validation of Molecular Subtypes

Consensus clustering categorized TCGA‐LUAD patients based on 33 ICD‐related genes into two subtypes. Principal component analysis (PCA) validated subtype separability using GEO datasets. Subtype classification consistency was further confirmed with consensus clustering and Nearest Template Prediction (NTP) from the MOVICS package.

### 2.4 Relationship Between ICD Subtypes and Clinical Features

Survival analysis compared outcomes between the identified subtypes in TCGA‐LUAD. Associations between subtypes and clinical characteristics were examined. The impact of clinicopathological features on OS in LUAD was evaluated using both univariate and multivariate Cox regression analyses.

### 2.5 Differences of ICD Genes, Driver Genes, and Their Mutations Between ICD Subtypes

A comparison of the 33 ICD‐related genes was conducted between two distinct subtypes, with further analysis of mutation profiles. Emphasis was placed on driver genes, pivotal in initiating and perpetuating molecular changes in tumors, to explore their intrinsic relationship with these subtypes and the association between mutations and subtype characteristics.

### 2.6 Relationship Between ICD Typing and TME

TME scores were calculated using the TMEscore package to compare differences between the two subtypes. TME signature scores were derived using the IOBR package. Immune cell abundance was assessed with CIBERSORT, and ESTIMATE scores were used to quantify stromal cell contributions.

### 2.7 ICD Typing With Immune Checkpoints and Drug Sensitivity

The relationship between ICD typing and immune checkpoints was analyzed. Immune infiltration levels of 29 cell types were assessed using ssGSEA. A heatmap illustrated the relationship between TME‐infiltrating cell frequencies and immune scores across the two ICD subtypes. Lastly, chemosensitivity profiles in LUAD subtypes were compared.

### 2.8 Association of IFNG With TME and Mutations

Eight genes from the analysis of differential expression among 33 genes associated with ICD in tumor and normal tissues were identified, with IFNG chosen for further investigation. Previous studies have noted that rapid production of type I interferons can activate TLR3 using cancer cell self‐RNA or trigger mtDNA release through the cGAS/STING pathway, enhancing the anticancer effects of certain chemotherapeutics [23, 24]. However, the breakdown of extracellular nucleic acids curtails the immunogenic response linked to regulated cell death. The impact of IFNG on ICD within tumor cells remains unexplored, necessitating further investigation. Associations of IFNG with immune cell populations, TME characteristics, and mutations were meticulously examined.

### 2.9 Cell Culture and Transfection

The following cell lines were used: BEAS‐2B (normal lung epithelium), A549, PC9, H1299 (human LUAD), and HUVEC from the National Collection of Authenticated Cell Cultures at the Precision Laboratory of the First Affiliated Hospital of Zhengzhou University. Cells were cultured at 37°C in a 5% CO_2_ environment using Dulbecco's Modified Eagle Medium (DMEM) enriched with 10% fetal bovine serum (Thermo Fisher Scientific, USA) and 1% penicillin–streptomycin (Thermo Fisher Scientific, USA). Plasmids overexpressing IFNG were constructed using the pcDNA3.1 vector and transfected using Lipofectamine2000 (Invitrogen, USA) reagent. For subsequent experiments, cells were collected 48 h post‐transfection.

### 2.10 RNA Isolation Accompanied by Real‐Time Quantitative PCR (RT‐qPCR)

Both normal and LUAD cells had their total RNA extracted with TRIzol reagent (Invitrogen, Carlsbad, CA, USA). Evo M‐MLV RT Premix was applied to generate complementary DNA, followed by RT‐qPCR utilizing the SYBR Green Premix Pro Taq HS qPCR Kit (Rox‐plus) (Accurate Biology, China). The internal reference gene utilized was β‐actin, with primer sequences provided in Table S1.

### 2.11 Western Blotting (WB)

LUAD cells were disrupted using RIPA buffer with Beyotime protease and phosphatase inhibitors. BCA detection kit was employed to assess protein concentrations. Using 10% SDS‐PAGE, identical protein amounts were separated and transferred to polyvinylidene fluoride membranes. Membranes were blocked for 2 h at ambient temperature with 5% skim milk in TBST, then stored overnight at 4°C with primary antibodies. Secondary antibodies conjugated to horseradish peroxidase (Proteintech) were applied for 2 h at ambient temperature, followed by visualization of protein bands using ECL chemiluminescence reagent. Primary antibodies included GAPDH (1:5000, #60004‐1‐Ig, Proteintech) and IFNG (1:1000, #15365‐1‐AP, Proteintech).

### 2.12 Immunohistochemical (IHC) Analysis for IFNG

Paraffin‐embedded LUAD tissue sections underwent deparaffinization, hydration, and antigen retrieval. Sections were stored throughout the night at 4°C using IFNG antibody (1:200, Proteintech), rinsed with PBS, and subsequently exposed to the secondary antibody at ambient temperature. Following further PBS rinses, sections were treated using DAB substrate and hematoxylin counterstain. Stained sections were examined and imaged using a 3DHISTECH panoramic scanning system. Image analysis for staining intensity was conducted using ImageJ software.

### 2.13 Immunofluorescence (IF)

Cells in 24‐well plates were seeded, allowed to adhere, and transfected with IFNG overexpression plasmid or control vector. After 48 h, cells were preserved with 4% paraformaldehyde for half an hour, treated using 5% BSA for 2 h at ambient temperature, then subjected to overnight incubation at 4°C with CRT primary antibody (1:200, #12238S, Cell Signaling Technology). On the following day, cells were exposed to fluorescent secondary antibody (A0423; Beyotime) for 2 h at ambient temperature in darkness. DAPI staining was performed for 20 min at room temperature, shielded from light. Fluorescence microscopy was used for observation and imaging.

### 2.14 Enzyme‐Linked Immunosorbent Assay (ELISA)

HMGB1 and ATP levels were quantified with ELISA assay kits (Elabscience) in accordance with the manufacturer's protocols. A microplate reader was utilized to assess absorbance at 450 nm.

### 2.15 Flow Cytometric Evaluation

Peripheral blood mononuclear cells (PBMCs) were extracted through density‐gradient separation, followed by the purification of CD14^+^ monocytes with CD14 MicroBeads. These cells were maintained in RPMI 1640 medium containing 10% FBS, rGM‐CSF, and IL‐4 for a duration of 5 days to facilitate DC differentiation. PBMCs co‐cultured with IFNG‐overexpressing or control cells were prepared for flow cytometry examination. Cells were labeled with Zombie Green for viability, followed by BV421‐αCD11c, PE‐αHLA‐DR, PE/Cyanine7‐αCD86, APC/Cyanine7‐αCD3, and PerCP/Cyanine5.5‐αCD8 for surface markers. Following fixation and permeabilization, cells were tagged using PE/Cyanine7‐αGranzyme B. Using a BD FACSCanto II flow cytometer, flow cytometry was executed, and FlowJo software was applied for the analysis of data.

### 2.16 Tube Formation Assay

Pre‐cooled extracellular matrix gel was dispensed into individual wells in a 96‐well format and set to gel for 30 min at 37°C. HUVECs were introduced at 1 × 10^4^ cells per well into conditioned medium and cultured for 6 h at 37°C with 5% CO_2_. Images of tube formation were taken using an inverted microscope (100× magnification). Quantitative analysis of tube formation was performed using ImageJ software.

### 2.17 Statistical Analysis

Analyses were performed using R version 4.2.0 along with appropriate packages. All analyses were conducted with a significance criterion of *p* < 0.05.

## 3 Results

### 3.1 Molecular Characteristics and Pathways of ICD‐Related Genes

**FIGURE 1:**
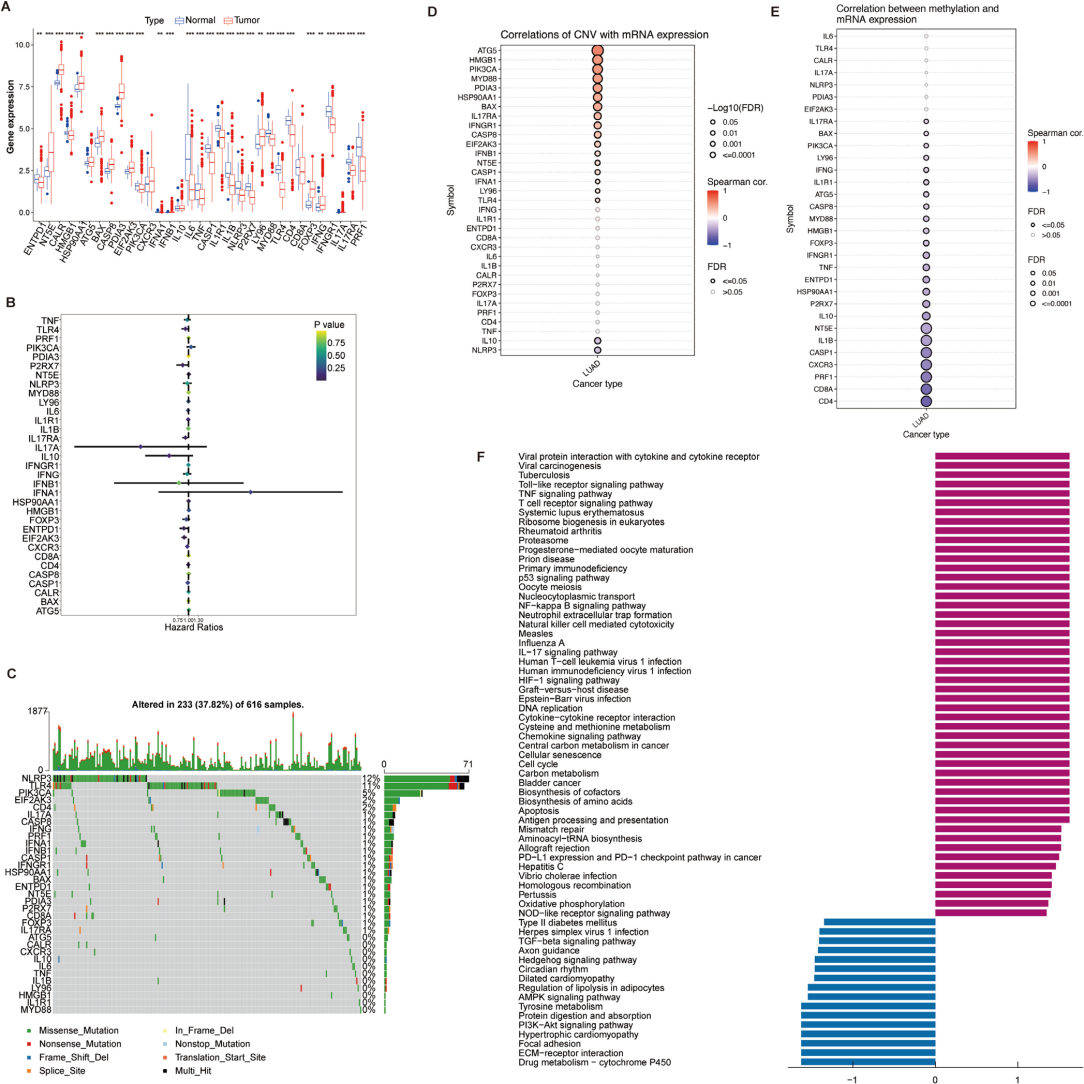
Detection of differentially expressed ICD‐related genes. (A) Comparative expression analysis of 33 ICD genes in tumor versus normal tissues. (B) Univariate Cox regression analysis assessing the prognostic implications of ICD‐related genes. (C) Waterfall plot illustrating the mutation frequencies of ICD‐related genes. (D, E) Correlations between ICD‐related genes and copy number variations (CNV) and methylation levels. (F) KEGG pathway analysis highlighting differences in pathways associated with positively and negatively correlated genes. *p < 0.05, **p < 0.01, ***p < 0.001.

We examined the expression profiles of 33 ICD genes in tumor versus normal tissues, revealing significant differential expression. NT5E, PDIA3, FOXP3, IL17A, and IFNG were upregulated, while PRF1, TLR4, and IL‐6 showed downregulation (Figure 1A). Univariate Cox regression using TCGA data indicated IL‐17A and IL‐10 as significant predictors of improved OS (Figure 1B). Mutation rates of ICD‐related genes in LUAD patients, notably NLRP3 (12%), were depicted in Figure 1C, and mutational signatures were illustrated in Figure S1A. Figure 1D,E explored correlations with CNV and methylation patterns. ssGSEA scores across samples were computed (Figure S1B). Kyoto Encyclopedia of Genes and Genomes (KEGG) pathway analysis highlighted associations with DNA replication, senescence, cell cycle regulation, apoptosis, programmed death ligand 1 (PD‐L1) expression, and the programmed death‐1 (PD‐1) pathway (Figure 1F).

### 3.2 Stratification and Prognostic Implications of ICD Gene Subtypes in LUAD Cohorts

**FIGURE 2:**
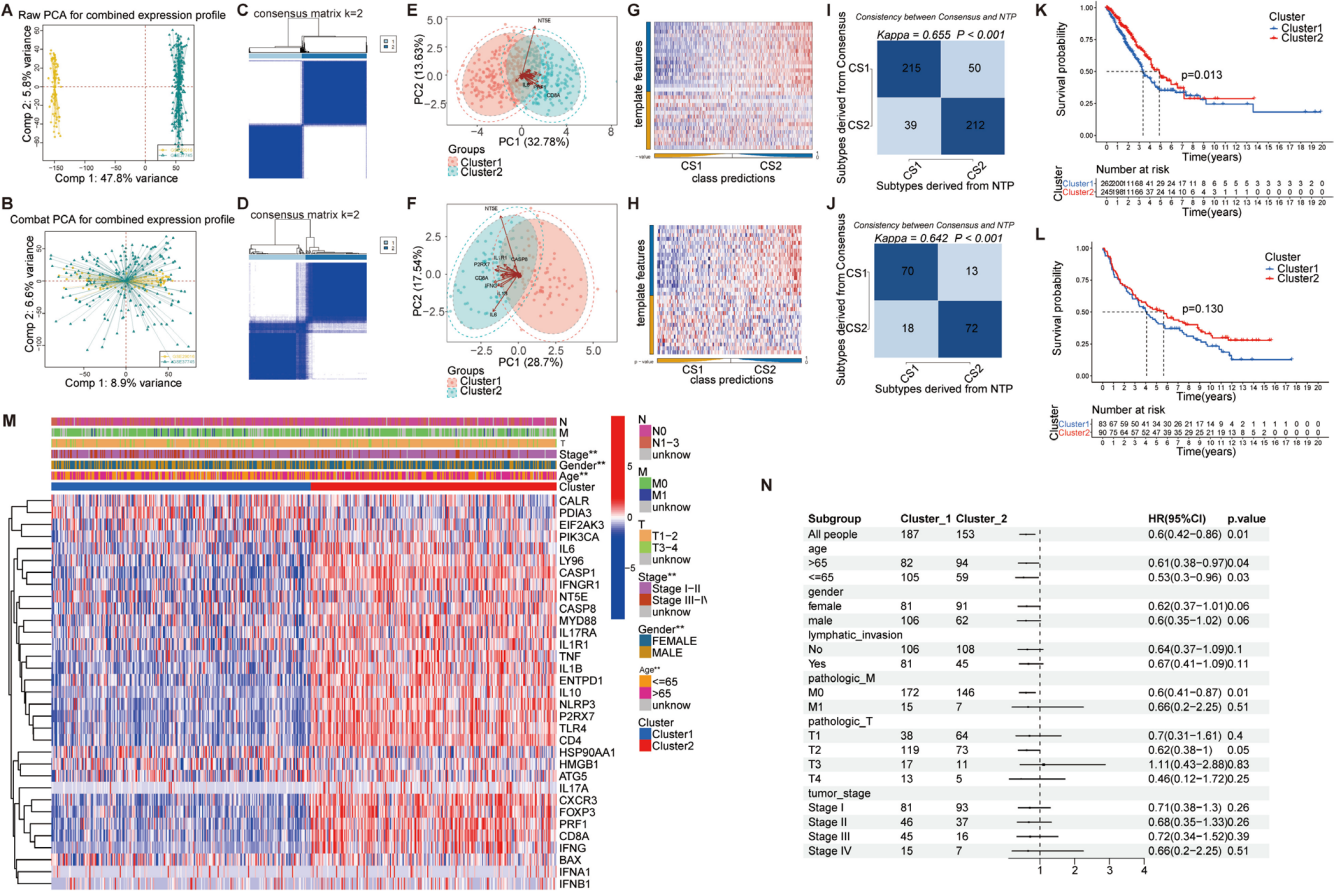
Consensus clustering and validation of ICD‐related molecular subtypes. (A, B) Raw and combat PCA for combined expression profile. (C, D) Consensus clustering in TCGA‐LUAD and GEO datasets. (E, F) PCA plots illustrating distinct clusters in TCGA‐LUAD and GEO datasets. (G–J) NTP identifying two subtypes in TCGA‐LUAD and GEO datasets. (K, L) Kaplan–Meier survival analysis comparing the two subtypes in TCGA and GEO cohorts. (M) Unsupervised clustering of 33 ICD‐related genes across all LUAD cohorts, annotated with patient characteristics including N, M, T stage, gender, age, and cluster. (N) Forest plot of multivariate Cox analysis showing the relationship between subtypes and prognosis, accounting for other clinical factors.

The sva package corrected batch effects in the GEO dataset. PCA showed gene expression distribution correction across four LUAD GEO cohorts before and after batch removal (Figure 2A,B). Using consensus clustering to classify ICD genes and validate them using the GEO dataset, two ICD gene types were identified (Figure 2C,D) and validated by PCA (Figure 2E,F). Subtype consistency was confirmed by NTP across TCGA‐LUAD and GEO datasets (*k* = 0.655, *p* < 0.001 for TCGA‐LUAD; *k* = 0.642, *p* < 0.001 for GEO) (Figure 2G–J).

Cluster 2 had a better prognosis than Cluster 1 in both datasets (Figure 2K,L). As illustrated in Figure 2M, cluster 2 demonstrated higher expression of ICD genes, suggesting a better prognosis associated with higher ICD gene expression. The multivariate Cox analysis showed that the relationship between subtypes and prognosis remained statistically significant after accounting for other clinical factors (Figure 2N). Significant differences in clinicopathologic characteristics between the two ICD subtypes were observed, including stage, gender, and age. Univariate and multivariate Cox regression analyses assessed clinical factors in OS using TCGA (346 samples, Figure S2). Univariate analysis showed significant differences in tumor stage, pathologic T stage, and lymphatic invasion in both TCGA‐LUAD and GEO cohorts. In multivariate analysis, pathologic T stage and lymphatic invasion were significantly associated with prognosis in TCGA‐LUAD, while tumor stage was significant in GEO.

### 3.3 Differential Expression and Mutation Patterns of ICD Subtypes

**FIGURE 3:**
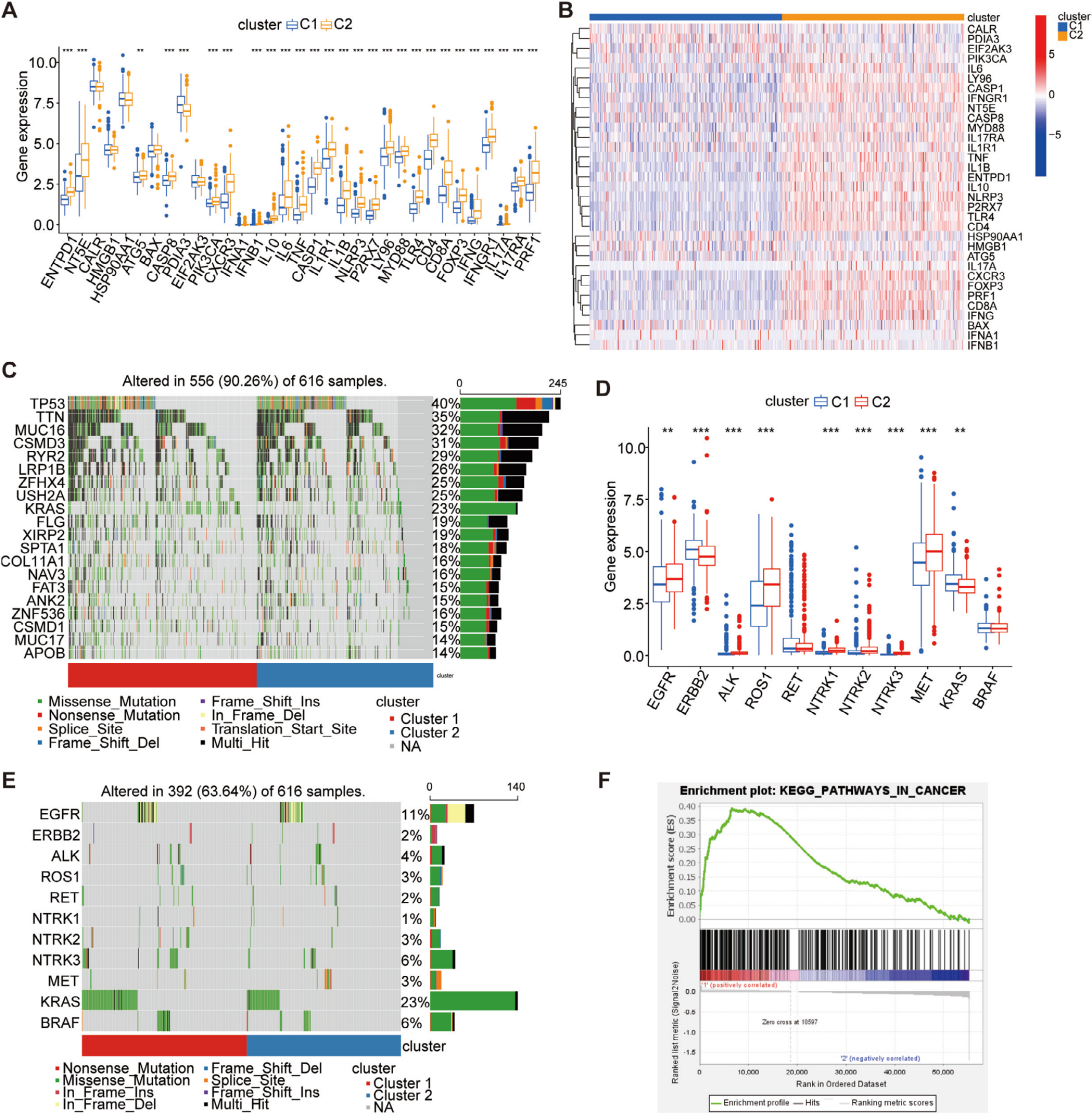
Expression and mutation contrasts across ICD subtypes. (A) Box plot illustrating the expression levels of 33 ICD‐related genes across two clusters. (B) Heat map depicting the expression patterns of 33 ICD‐related genes in the two clusters. (C) Waterfall plot illustrating the differences in the gene mutation landscape between the two clusters. (D) Box plot showing the differential expression of driver genes between the two subtypes. (E) Waterfall plot illustrating the differences in the mutation landscape of driver genes between the two clusters. (F) Gene Set Enrichment Analysis (GSEA) revealing the main enrichment pathways.

We analyzed expression differences in 33 ICD‐related genes across two subtypes. Cluster 2 showed significantly higher expression levels compared to cluster 1 (Figure 3A,B). TP53 was prominently mutated in both subtypes (Figure 3C). As for driver genes, cluster 2 predominantly expressed EGFR, ALK, ROS1, NTRK1, NTRK2, NTRK3, and MET, while KARS and ERBB2 were more expressed in cluster 1 (Figure 3D). KARS had the highest mutation rate across both clusters (Figure 3E). GSEA indicated a significant enrichment of PATHWAY IN CANCER (Figure 3F).

### 3.4 TME Profiling and Chemotherapy Sensitivity in ICD Subtypes

**FIGURE 4:**
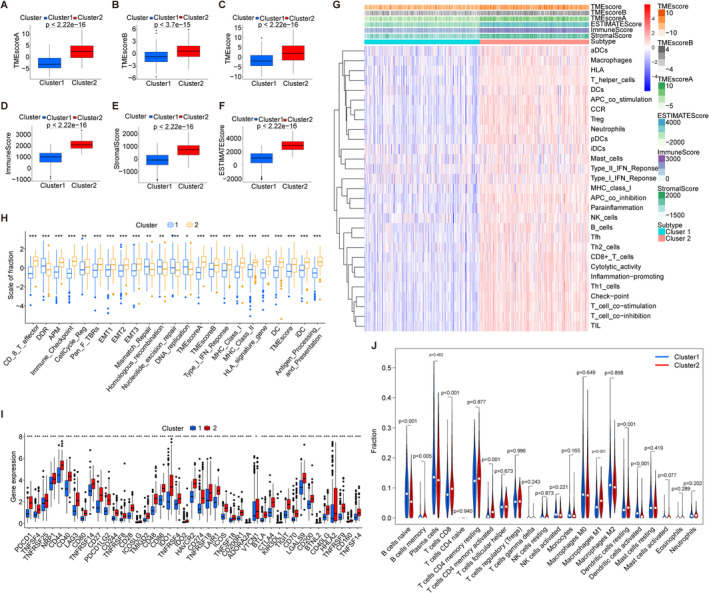
Comprehensive analysis of TME characteristics and immune‐related factors in two subtypes. (A–F) Box plots illustrating differences in TMEscoreA, TMEscoreB, TMEscore, Immune Score, Stromal Score, and ESTIMATE Score between the two groups. (G) Heat map depicting differential expression of immunocyte markers, Immune score, Stromal score, ESTIMATE score, and TME score. (H) Box plot showing the distribution of TME‐related signatures across two subtypes. (I) Differential expression of immune‐related genes between the two groups. (J) Comparison of TME cell infiltration abundance between the two groups.

TME crucially regulates tumorigenesis, metastasis, and immunotherapy response [25, 26, 27]. TMEscore serves as a prognostic biomarker in LUAD, correlating higher scores with improved survival [28]. Figure 4A–C shows TMEscoreA, TMEscoreB, and overall TMEscore differences between subtypes, with cluster 2 consistently higher. Cluster 2 also exhibits elevated Immune, Stromal, and ESTIMATE scores compared to cluster 1 (Figure 4D–F). A heatmap highlights increased immune cell expression in cluster 2, indicating a more immunogenic TME (Figure 4G). TME‐related signatures such as CD8^+^ T effector cells, immune checkpoints (e.g., PDCD1, CTLA4), MHC class 1, and DCs are notably higher in cluster 2 (Figure 4H,I). Differences in immune cell abundance further distinguish the subtypes, with cluster 2 showing higher levels of CD8^+^ T cells and activated memory CD4^+^ T cells, while cluster 1 exhibits higher levels of naive B cells (Figure 4J). Comparative drug sensitivity analysis reveals cluster 2's higher sensitivity to cisplatin, paclitaxel, and etoposide, underscoring potential therapeutic implications (Figure S3).

### 3.5 Analysis Revealed Significant Differential Expression of IFNG in LUAD

**FIGURE 5:**
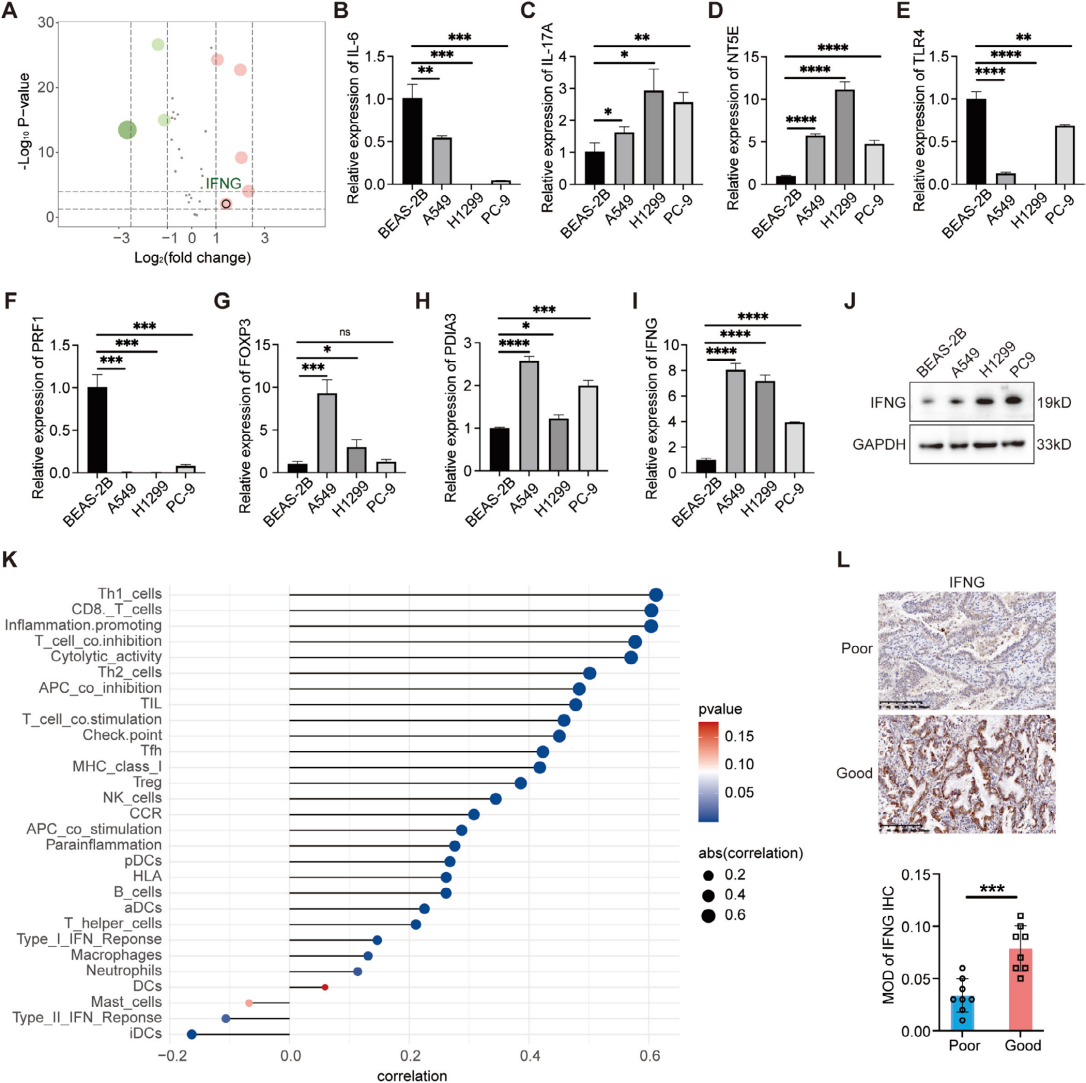
Integrated analysis of ICD gene expression, IFNG regulation, and prognostic significance in LUAD. (A) Volcano plot of differentially expressed genes. (B‐I) RT‐qPCR validation of 8 ICD genes in normal lung epithelial cell lines and human LUAD cell lines. (J) Western blot analysis of IFNG protein expression in normal lung epithelial cell lines and human LUAD cell lines. (K) Bubble plot illustrating the correlation between IFNG expression and immune cell infiltration levels. (L) IHC staining and MOD calculating of IFNG between LUAD patients with good (*n* = 8) and poor prognosis (*n* = 8). *p < 0.05, **p < 0.01, ***p < 0.001, ****p < 0.0001.

Our analysis identified significant differential expression of eight ICD‐related genes between normal lung tissues and LUAD samples: NT5E, PDIA3, IL6, TLR4, FOXP3, IFNG, IL17A, and PRF1 (Figure 5A). RT‐qPCR validation confirmed the consistent expression patterns of these genes in both normal lung epithelial cells and LUAD cells, consistent with our initial findings (Figure 5B–I). Given the unclear relationship between IFNG and the induction of anticancer effects through ICD, we further investigated its role. WB analysis validated IFNG expression at the protein level, corroborating the RT‐qPCR results (Figure 5J). Analysis of the correlation between high mutational burden and IFNG expression revealed TP53 as the most highly correlated gene (Figure S4A). IFNG exhibited positive correlations with most immune‐related signature scores but a negative correlation with TMEscoreB (Figure S4B). Bubble plots demonstrated that IFNG expression positively correlated with levels of immune cell infiltration, particularly with Th1 cells and CD8 T cells (Figure 5K). To further explore the impact of IFNG, IHC was performed on LUAD patient tissues, revealing higher IFNG expression in tissues from patients with a favorable prognosis compared to those with a poor prognosis (Figure 5L). These findings underscore the potential significance of IFNG in the immune response within the tumor microenvironment of LUAD, suggesting its role as a potential biomarker for prognosis and immunotherapy response in LUAD.

### 3.6 Impact of IFNG Expression on ICD and Angiogenesis in LUAD

**FIGURE 6:**
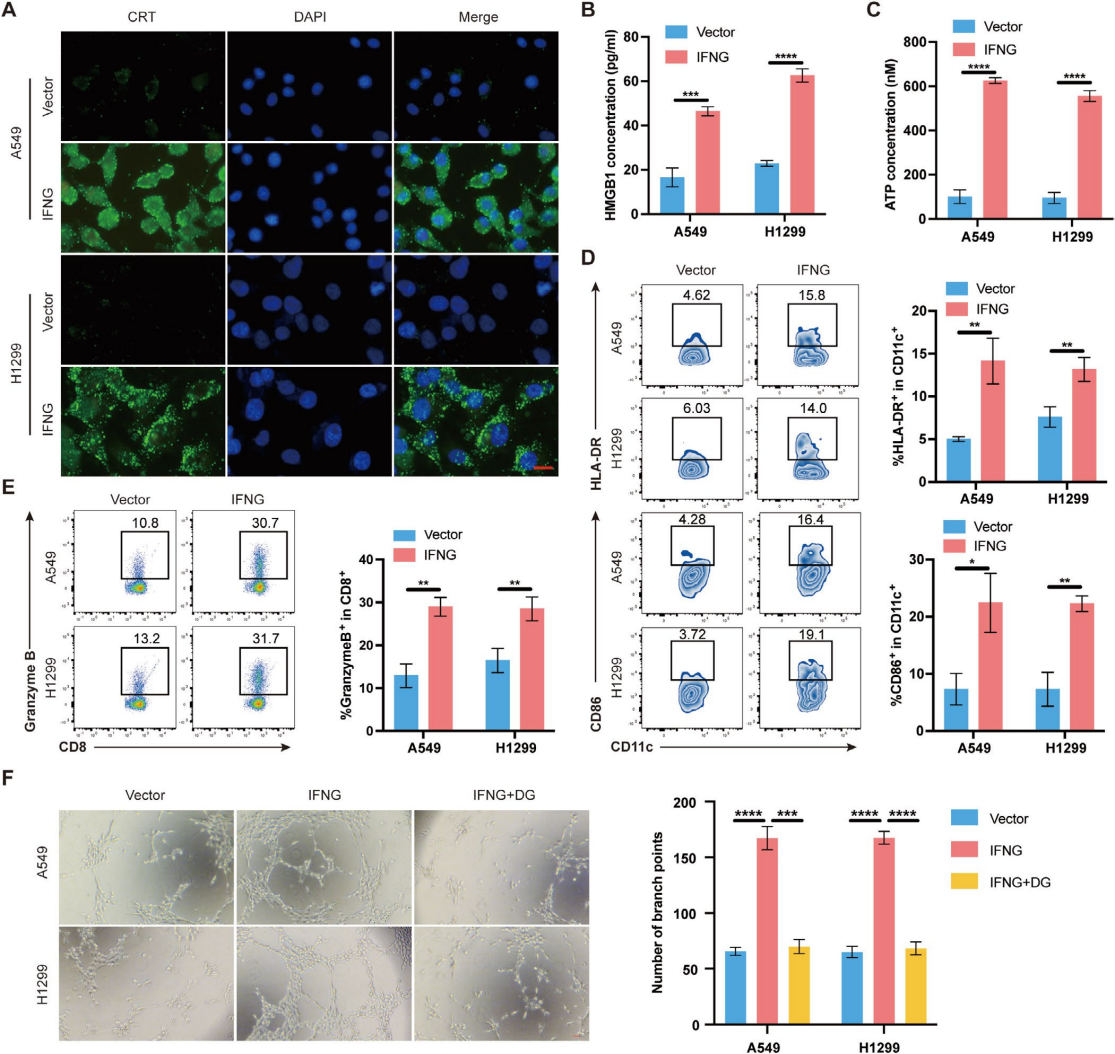
IFNG in LUAD cells induces ICD and promotes angiogenesis. (A) Immunofluorescence analysis of cells stained for CRT and DAPI. Scale bar: 20 μm. (B) ELISA detecting secreted HMGB1. (C) ELISA detecting secreted ATP. (D) Flow cytometry detecting the expression of CD86 and HLA‐DR on DCs surface. (E) Flow cytometry detecting the expression of Granzyme B on CD8^+^ T cell surface. (F) Tube formation assay demonstrating the effect of IFNG expression in LUAD cells on HUVECs. Scale bar: 100 μm. *p < 0.05, **p < 0.01, ***p < 0.001, ****p < 0.0001.

In order to investigate the role of IFNG expression in tumor cells regarding ICD, we generated IFNG control and overexpressing cell lines using A549 and H1299 LUAD cell lines. Assessment of ICD induction included evaluation of CRT exposure, HMGB1 release, and ATP secretion as described by Krysko et al. [13]. If analysis revealed significant CRT exposure on the surface of A549 and H1299 cells with IFNG overexpression compared to controls (Figure 6A). Moreover, IFNG overexpression markedly enhanced HMGB1 release and ATP secretion in these cells (Figure 6B,C), indicating increased exposure or release of DAMPs. To explore the impact of DAMPs exposure on TME, PBMCs co‐cultured with IFNG control or overexpressing A549 and H1299 cells were analyzed using flow cytometry. The expression of HLA‐DR and CD86 on DCs significantly increased in the IFNG group (Figure 6D), along with elevated secretion of Granzyme B by CD8^+^ T cells (Figure 6E). These results indicate that overexpression of IFNG promotes DC maturation and enhances the cytotoxic function of CD8^+^ T cells. Given previous findings suggesting a role of HMGB1 in tumor angiogenesis [29], we further investigated the influence of IFNG expression on this process. Experimental results showed that IFNG overexpression enhanced tube formation in HUVECs, an effect that was reversed upon the addition of the HMGB1 inhibitor dipotassium glycyrrhizinate (DG) (Figure 6F). Collectively, our study indicates that IFNG overexpression in tumor cells induces ICD, promotes DC maturation, and enhances CD8^+^ T cell function. Additionally, it enhances angiogenesis, with this effect being reversible through HMGB1 inhibition.

## 4 Discussion

ICD, a process primarily observed in cancer cells, has gained significant recognition recently [30]. It induces DAMPs release, promoting antigen presentation by DCs and CD8^+^ T cell proliferation, thereby activating the host immune response against cancer [12, 31]. For instance, CRT serves as a marker of ICD, enhancing DC antigen‐presenting capacity by acting as an “eat me” signal [32]. Extracellular HMGB1 activates DCs and aids in tumor‐associated antigen processing through TLR4 binding [33, 34]. These immunostimulatory signals activate T cells, leading to their infiltration into tumors and subsequent elimination of residual cancer cells [35]. Consequently, ICD has emerged as pivotal in cancer therapy.

In this study, we identified 33 ICD‐related genes, many of which showed significant differential expression between tumor and normal tissues. Cox regression analysis indicated that IL‐17A and IL‐10 are prognostic indicators associated with improved OS. NLRP3 emerged as the most frequently mutated gene among ICD‐related genes in LUAD, impacting immune response, apoptosis, and tumorigenesis [36]. Co‐mutations were common across these genes. KEGG pathway analysis revealed associations with key pathways including DNA replication, cellular senescence, cell cycle regulation, apoptosis, and the PD‐L1/PD‐1 checkpoint pathway. Given LUAD's heterogeneity, we classified samples into two molecular subtypes based on these genes, validated independently. These subtypes differed significantly in prognosis, clinicopathologic features, mutation profiles, tumor microenvironment, immune infiltration, and drug response. Cluster 2, characterized by higher ICD gene expression, showed a more favorable prognosis than Cluster 1. Our findings suggest that increased ICD gene expression may correlate with better outcomes in LUAD, highlighting potential implications for personalized treatment strategies.

In addition, we explored the differential expression of driver genes across these subtypes. Cluster 2 showed higher expression of key drivers such as EGFR, ALK, ROS1, NTRK1, NTRK2, NTRK3, and MET, whereas KARS and ERBB2 were predominantly expressed in cluster 1. These driver genes are pivotal in targeted anticancer therapies and serve as prognostic biomarkers [37]. Mutational analysis identified KARS as the most frequently mutated driver gene in both clusters, known for its role in regulating tumor cell processes [38]. GSEA underscored significant enrichment in cancer‐related pathways.

Furthermore, we investigated the association between ICD subtypes and the TME. Cluster 2 consistently exhibited higher scores across various TME metrics (TMEscoreA, TMEscoreB, TMEscore, Immune score, Stromal score, and ESTIMATE score) compared to cluster 1. TME‐related signatures including CD8^+^ T effector cells and immune checkpoint markers varied significantly between clusters. Notably, cluster 2 displayed elevated levels of CD8^+^ T cells and activated memory CD4^+^ T cells, contrasting with cluster 1, which showed a predominance of naive B cells. The profound influence of TME on tumor behavior and therapeutic response is well established [39]. Gene expression analysis revealed that cluster 2 displayed higher expression of immune checkpoint molecules such as PDCD1 and Recombinant Cytotoxic T‐Lymphocyte Associated Antigen 4 (CTLA4), suggesting a potential advantage for immunotherapy. PDCD1 encodes the PD‐1 protein, a key regulator of T‐cell function that interacts with PD‐L1 expressed on cancer cells' surfaces [40]. Antibodies targeting PD‐1 or PD‐L1 have shown efficacy in various cancers, including non‐small‐cell lung cancer (NSCLC) [41, 42]. In contrast, CTLA4 influences early immune responses and regulatory T cell‐mediated immunosuppression [43]. Additionally, comparative analysis of chemotherapy drug sensitivity indicated that cluster 2 exhibited enhanced responsiveness to cisplatin, paclitaxel, and etoposide.

Additionally, we identified eight genes among a panel of 33 ICD‐related genes showing differential expression between tumor and normal tissues. NT5E, PDIA3, FOXP3, IL17A, and IFNG were upregulated, whereas PRF1, TLR4, and IL‐6 were downregulated. Notably, NT5E (CD73) was markedly overexpressed in NSCLC, correlating with enhanced cell growth, cycle progression, and migration, regulated in part by miR‐30a‐5p [44]. PDIA3 mutations have implications in lung cancer via abnormal immunosurveillance [45], while FOXP3 acts as a co‐activator in NSCLC, promoting Wnt/β‐catenin signaling, epithelial‐mesenchymal transition, and metastasis [46]. IL17A contributes to angiogenesis and metastasis in NSCLC [47]. PRF1 enhances cytotoxicity through granzyme uptake [48, 49], and TLR4 and IL‐6 are associated with inflammation and poor outcomes [50, 51]. IFNG, crucial in immunity, correlates significantly with TP53 mutation and immune cell types, notably CD8^+^ T cells.

IFNG derived from T cells has been reported to predict the efficacy of immune checkpoint inhibitors (ICIs) [52]; however, the role of IFNG within tumor cells has not yet been documented. Our study validated IFNG's expression and protein levels in tumor cells. IHC in LUAD patients revealed higher IFNG in favorable prognosis cases, suggesting its prognostic potential. As an ICD‐related gene, IFNG overexpression in A549 and H1299 cells induced the occurrence of ICD, promoted CRT exposure, HMGB1 release, and ATP secretion, subsequently promoting DCs maturation and enhancing CD8^+^ T cell function. Nuclear HMGB1 can support IFNG expression in CD8^+^ T cells by directly regulating the activity of its transcription factors [53]. Additionally, HMGB1 released during the ICD of tumor cells promotes IFNG secretion by CD8^+^ T cells [54]. In lung cancer cells treated with IFNG, both transcription and secretion of HMGB1 are increased [55]. Our study found that overexpression of IFNG in LUAD cells induces ICD, thereby promoting HMGB1 release. Dead tumor cells release ATP, a danger signal that stimulates antigen presentation by dendritic cells, leading to the activation of IFNG‐producing T cells [56, 57]. Our study demonstrates that increased intracellular IFNG in tumor cells can induce ICD, thereby promoting ATP release.

The important role of IFNG in cancer therapy has been thoroughly validated in numerous clinical trials and animal studies. The use of IFNG may enhance chemotherapy's effects by increasing tumor‐associated antigen release from dying cells, thereby strengthening the immune response against tumors [58]. IFNG treatment can enhance tumor antigen presentation and promote T cell infiltration in the TME, transforming cold tumors into hot tumors [59]. Icaritin can elevate IFNG levels and significantly inhibit tumor growth in combination with ICIs [60]. However, the role of IFNG in tumor cells is rarely reported. Our study shows that increasing intracellular IFNG levels can induce ICD, enhancing immune cell recognition and killing of tumor cells.

The inhibitory effect of IFNG on tumor angiogenesis has been widely reported. For instance, Professor Blankenstein found that IFNG can act on endothelial cells within tumors, promoting tumor vascular contraction and disrupting the tumor's energy supply, ultimately leading to tumor regression [61]. However, Yan Liu et al. indicated that IFNG can increase the expression of HIF‐1α in mesenchymal stem cells, thereby upregulating VEGF expression and promoting tumor angiogenesis [62]. Our research demonstrated that IFNG facilitated tumor angiogenesis via HMGB1 pathways, mitigated by HMGB1 inhibition.

In conclusion, using 33 ICD‐related gene expression profiles, we categorized TCGA LUAD samples into two distinct subtypes with significant differences in prognosis, gene mutations, TME, TMB, immune cell infiltration, and drug sensitivity. Elevated IFNG expression in LUAD enhances CRT exposure, HMGB1, and ATP release, augmenting ICD and promoting DC maturation and CD8^+^ T cell function. Concurrently, IFNG overexpression promotes angiogenesis, a process reversible by HMGB1 inhibitors. The identification of novel molecular subtypes based on ICD‐related genes holds promise for guiding personalized therapies in LUAD, assessing prognosis, and predicting immunotherapy efficacy. Moreover, IFNG emerges as a potential prognostic biomarker and therapeutic target in LUAD, exerting dual effects on tumor microenvironment modulation and tumor angiogenesis. These findings provide novel insights into therapeutic strategies targeting IFNG‐mediated pathways in LUAD patients.

## Author Contributions

**Lifeng Li:** conceptualization (equal), funding acquisition (equal), investigation (equal), methodology (equal), project administration (equal). **Yaqi Yang:** conceptualization (equal), data curation (equal), investigation (equal), methodology (equal), writing – original draft (equal). **Mengle Peng:** conceptualization (equal), data curation (equal), investigation (equal). **Biyue Wang:** investigation (equal). **Lili Zhu:** resources (equal). **Chengxin Chen:** writing – original draft (equal). **Zhirui Fan:** resources (equal). **Xiaoran Duan:** resources (equal). **Ruyue Xue:** resources (equal). **Xuefeng Lv:** resources (equal). **Ming Cheng:** resources (equal). **Jie Zhao:** conceptualization (equal), funding acquisition (equal), project administration (equal), writing – review and editing (equal).

## Ethics Statement

The research protocol was approved by the First Affiliated Hospital of Zhengzhou University.

## Consent

The authors have nothing to report.

## Conflicts of Interest

The authors declare no conflicts of interest.

## Supporting information


**Figure S1.** (A) Mutational signatures of the 33 ICD‐related genes, with green indicating co‐mutations, brown‐green indicating specific co‐mutations, and brown indicating individual mutations. (B) Single‐sample Gene Set Enrichment Analysis (ssGSEA) scores for each gene.

**Figure S2.** Univariate and multivariate Cox regression analysis of clinical characteristics with OS in both cohorts.

**Figure S3.** IC50 levels of chemotherapy drugs in the two subtypes.

**Figure S4.** (A) Heat map showing correlation between high mutation genetic variation and IFNG expression. (B) Spearman’s correlation analysis of immune‐related signature scores with IFNG expression.

Table S1.

## Data Availability

The data were downloaded from TCGA and GEO databases. Other raw data and materials can be made available upon reasonable request to the corresponding author.
