# Supplementary figures and images for: Molecular Subtyping and Therapeutic Targeting of IFNG‐Driven Immunogenic Cell Death in Lung Adenocarcinoma
Source: Cancer Med. 2025 Feb 13;14(4):e70678. doi: 10.1002/cam4.70678 (PMC11822994; doi:10.1002/cam4.70678)

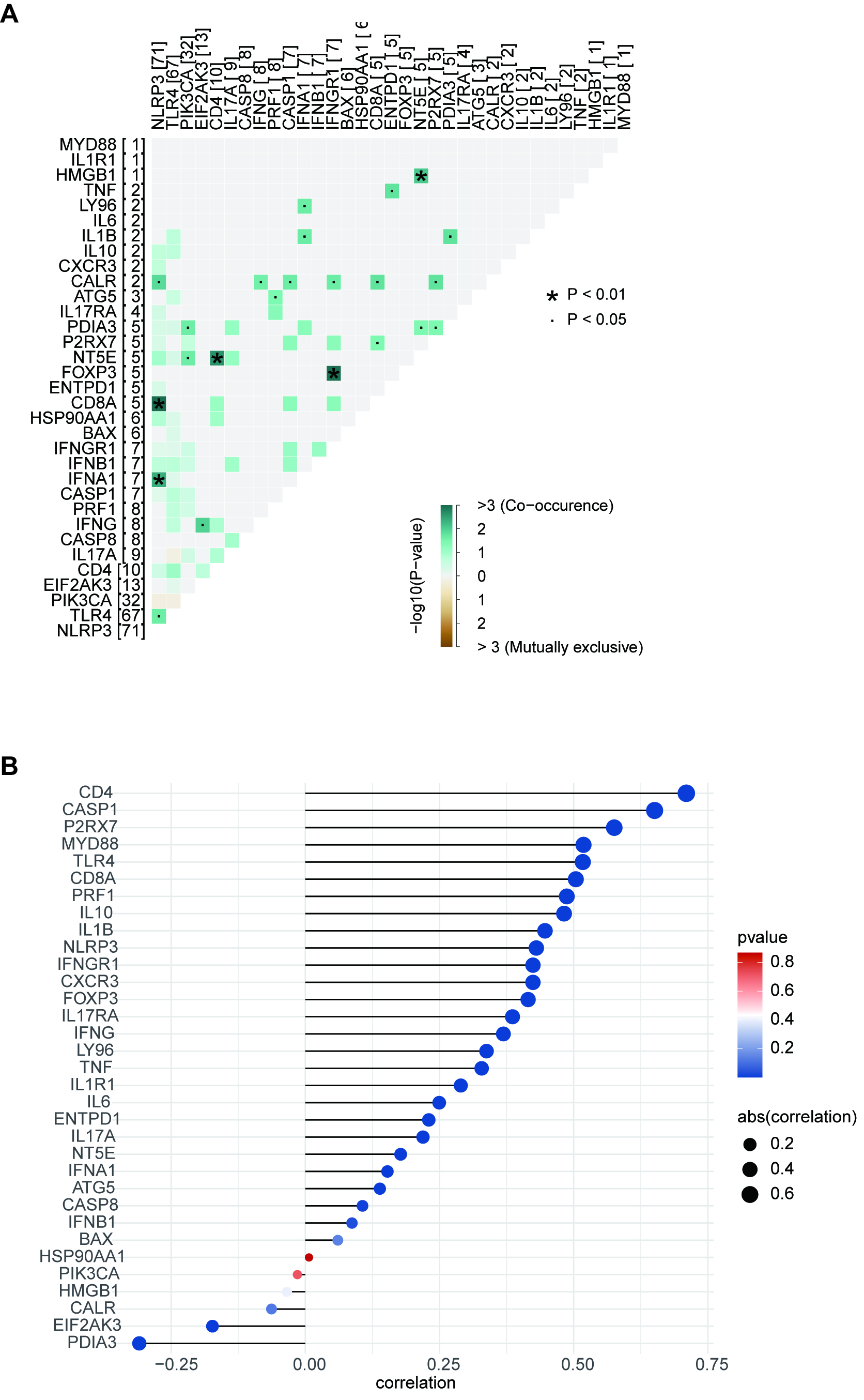

Supplement: Supplementary file 1 — Figure S1. (A) Mutational signatures of the 33 ICD‐related genes, with green indicating co‐mutations, brown‐green indicating specific co‐mutations, and brown indicating individual mutations. (B) Single‐sample Gene Set Enrichment Analysis (ssGSEA) scores for each gene. [file CAM4-14-e70678-s004.tif]

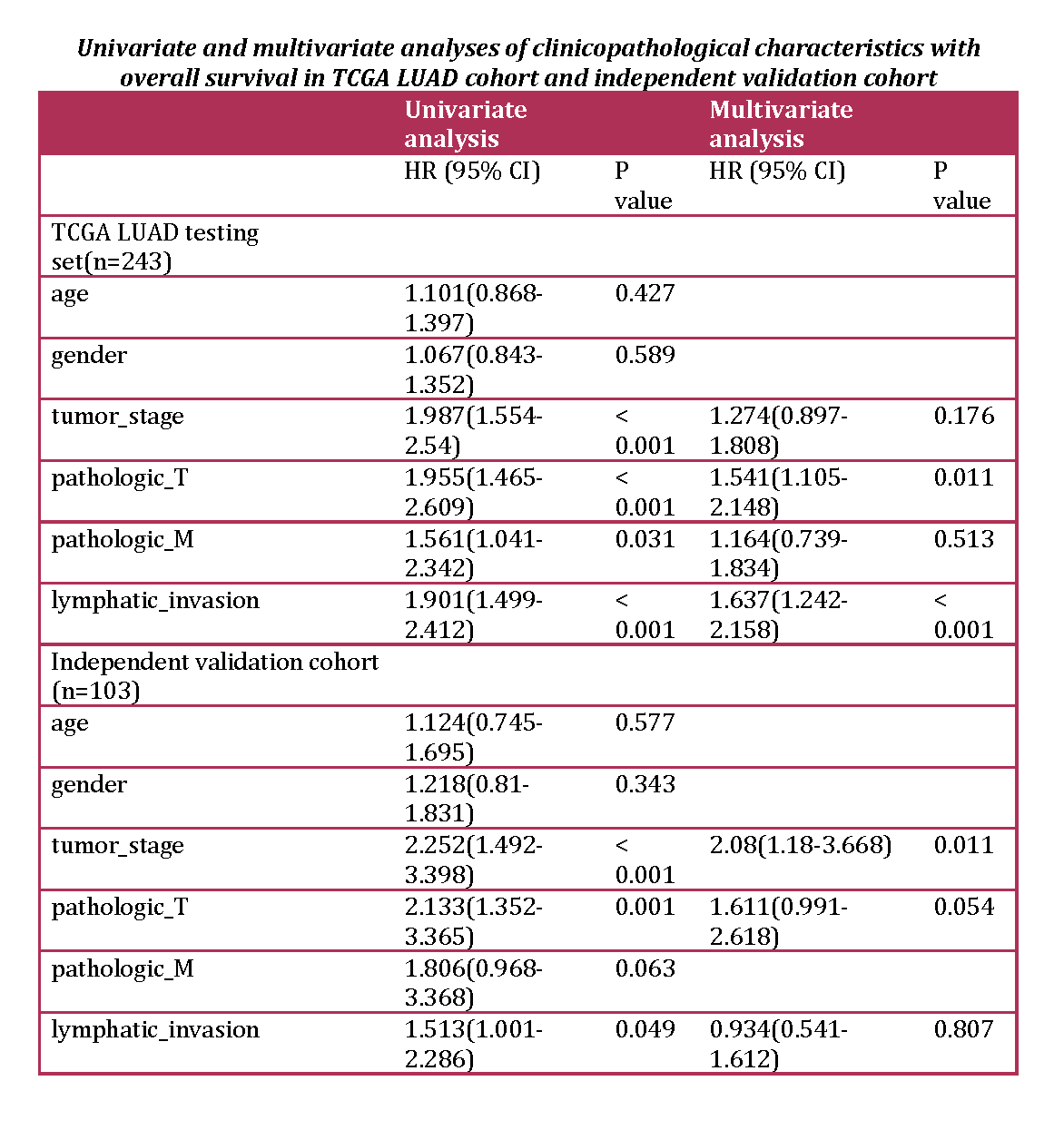

Supplement: Supplementary file 2 — Figure S2. Univariate and multivariate Cox regression analysis of clinical characteristics with OS in both cohorts. [file CAM4-14-e70678-s002.tif]

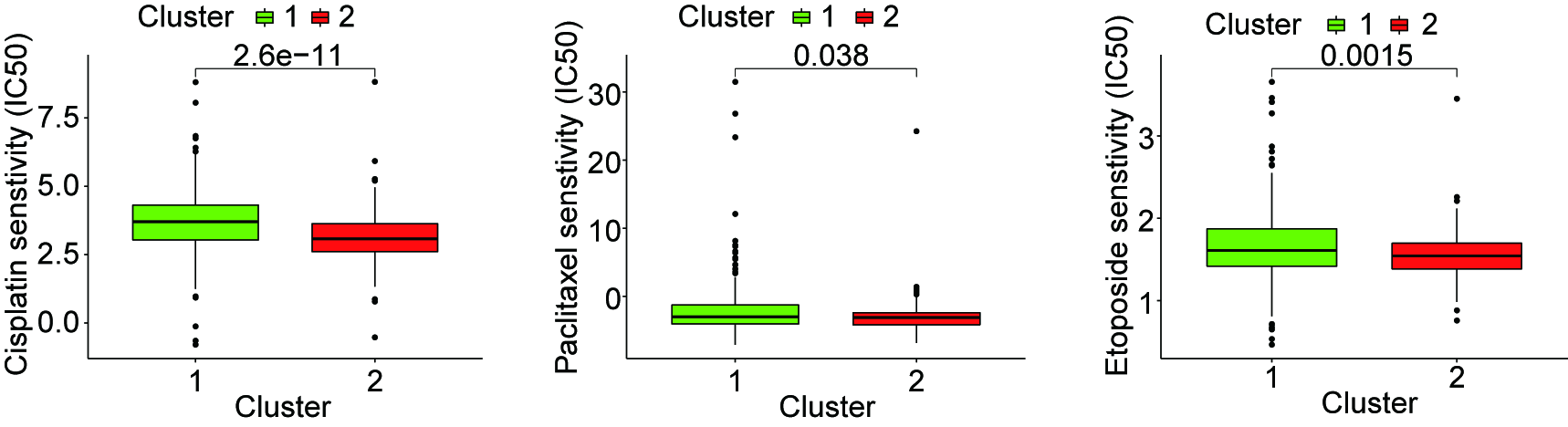

Supplement: Supplementary file 3 — Figure S3. IC50 levels of chemotherapy drugs in the two subtypes. [file CAM4-14-e70678-s005.tif]

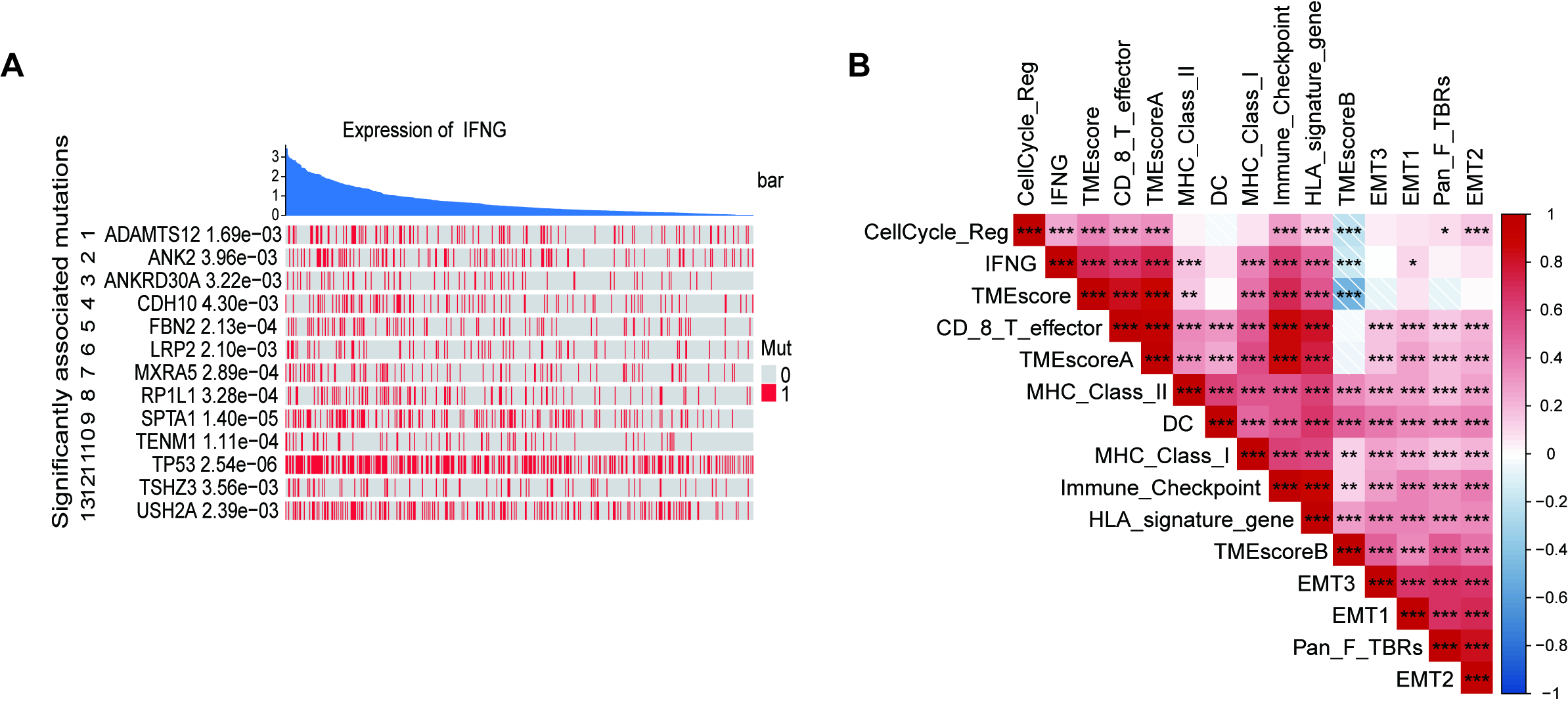

Supplement: Supplementary file 4 — Figure S4. (A) Heat map showing correlation between high mutation genetic variation and IFNG expression. (B) Spearman’s correlation analysis of immune‐related signature scores with IFNG expression. [file CAM4-14-e70678-s001.tif]
